# Prophylactic Hyperthermic Intraperitoneal Chemotherapy for Patients at High Risk of Developing Gallbladder Cancer Peritoneal Metastases: Case Report and Rationale for a Prospective Clinical Trial

**DOI:** 10.3390/jcm13030768

**Published:** 2024-01-29

**Authors:** Alexander E. Crum, Michael Sestito, Mary Garland-Kledzik, Brian A. Boone

**Affiliations:** 1 School of Medicine, West Virginia University, Morgantown, WV 26506, USA; crumalexandere@gmail.com; 2 Department of Surgery, West Virginia University, Morgantown, WV 26506, USA; michael.sestito@hsc.wvu.edu (M.S.); molly.kledzik@hsc.wvu.edu (M.G.-K.)

**Keywords:** gallbladder cancer, hyperthermic intraperitoneal chemotherapy (HIPEC), peritoneal, prophylactic

## Abstract

Gallbladder cancer is a devastating disease with a 5-year survival of only 18%. The majority of gallbladder cancers are discovered incidentally in patients undergoing cholecystectomy. During non-oncologic laparoscopic cholecystectomy for gallbladder disease, gallbladder perforation occurs in 29% of cases and spillage of gallstones occurs in 9% of cases. Patients with gallbladder cancer frequently develop peritoneal recurrence, particularly after intra-operative bile spillage during cholecystectomy for incidental gallbladder cancer. The high likelihood of spillage and peritoneal seeding during cholecystectomy for incidental gallbladder cancer suggests the need for prophylactic strategies to prevent peritoneal carcinomatosis. Hyperthermic intraperitoneal chemotherapy (HIPEC) has efficacy in gallbladder cancer patients with macroscopic peritoneal disease undergoing cytoreductive surgery and has been associated with a survival advantage in a multi-institutional retrospective case series. However, the utilization of HIPEC with a prophylactic intent against the development of peritoneal disease following resection of gallbladder cancer has not yet been prospectively studied. Here, we review the literature surrounding gallbladder cancer and HIPEC, report an institutional experience utilizing prophylactic HIPEC, and discuss a recently proposed prospective clinical trial evaluating the efficacy of prophylactic HIPEC in the prevention of gallbladder peritoneal metastasis.

## 1. Introduction

Gallbladder cancer (GBC) had an estimated worldwide incidence of 115,949 new cases, and 84,695 new deaths in 2020 [1]. GBC is the most common biliary tract cancer worldwide; however, it remains relatively uncommon in the United States, with an incidence of fewer than 4000 new cases annually. This makes both retrospective studies and randomized control trials evaluating treatments for this disease particularly difficult. The 5-year survival for all patients with GBC is 18%, and survival falls to nearly 0% for patients with metastatic disease [2]. The majority of patients succumb to metastatic disease, with a significant number developing peritoneal carcinomatosis. As a result, there is a critical need to improve our understanding of the pathophysiology of peritoneal metastasis to optimize the treatment and prevention of recurrent disease at this site. The identification of high-risk patient populations and the development of innovative treatment options to combat this aggressive disease are important goals. 

The concept of prophylactic heated intraperitoneal chemotherapy (HIPEC) as a treatment for preventing peritoneal recurrence has been introduced and studied over the past decade. Initial results from randomized controlled trials (RCTs) in more prevalent gastrointestinal (GI) malignancies call for cautious optimism in this strategy as part of a multimodal treatment sequence [3,4,5,6,7,8]. Therapeutic HIPEC in GBC patients with macroscopic peritoneal disease undergoing cytoreductive surgery (CRS) has been associated with a survival advantage in a multi-institutional retrospective case series [9]. These studies suggest that there may be utility for prophylactic HIPEC in GBC patients as well. The objectives of this article are to explore the literature surrounding gallbladder cancer and HIPEC, review the patterns of treatment failure in gallbladder cancer, report an institutional experience of utilizing prophylactic HIPEC, and discuss a recently proposed clinical trial evaluating the feasibility of prophylactic HIPEC in the prevention of gallbladder metastasis. Here, we conduct a nonsystematic review of the literature surrounding gallbladder cancer and HIPEC.

Case: A 73-year-old male presented to the local emergency department with severe acute right upper quadrant abdominal pain and was diagnosed with acute cholecystitis. He underwent a laparoscopic cholecystectomy with spillage of gallstones and bile during the procedure. Pathological examination revealed a well-differentiated invasive adenocarcinoma staged pT2a. The cystic duct and liver parenchymal margins were uninvolved by the invasive carcinoma. The patient was referred to our institution for further management. 

**Figure 1:**
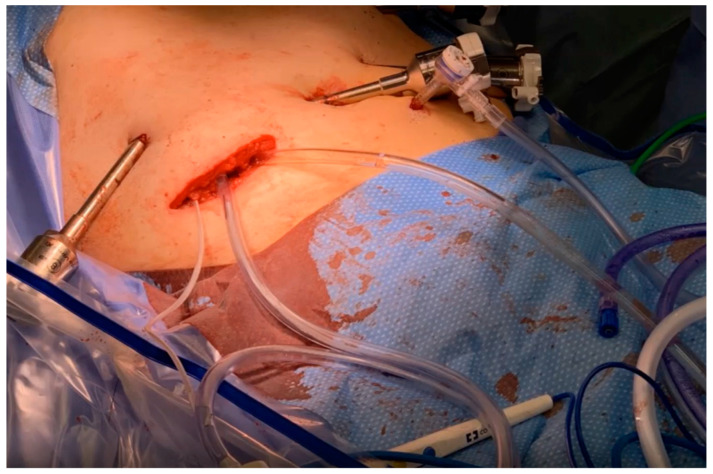
Minimally invasive, prophylactic HIPEC was utilized following robotic assisted central hepatectomy and portal lymphadenectomy in a patient at high risk of peritoneal recurrence due to intra-operative bile and gallstone spillage. The specimen extraction site was used to place the inflow and outflow catheters and tissue temperature probe.

The patient was offered a robotic central hepatic resection and portal lymphadenectomy as standard of care for GBC. Given the intra-operative bile and stone spillage during the index operation, he was deemed at high risk for peritoneal recurrence and, therefore, offered an omentectomy and prophylactic HIPEC as part of a pilot clinical trial protocol. After the oncologic resection, chemotherapy was administered through perfusion inflow and outflow catheters placed through the specimen extraction site and laparoscopically positioned (Figure 1). HIPEC was performed for 100 min using 40 mg of mitomycin C (MMC) heated to 42 °C. The abdomen was then irrigated with 4 L of saline and was washed out utilizing a laparoscopic suction irrigator. 

The patient tolerated the treatment well. His postoperative course was uncomplicated, and he was discharged home on post-operative day 4. He is currently doing well 4 years post-operatively and surveillance imaging continues to show no evidence of disease. This case demonstrates the feasibility of prophylactic HIPEC for patients with GBC at high risk for peritoneal seeding.

## 2. Discussion

### 2.1. Clinical Presentation of Gallbladder Cancer

Patients with early invasive GBC are commonly asymptomatic or display nonspecific signs of gallbladder disease. The lack of specific symptoms is one of the factors that makes pre-operative diagnosis of GBC so challenging. The significant overlap of these symptoms with the presentation for acute cholecystitis and other benign biliary diseases confounds the diagnosis. Risk factors for GBC, such as primary sclerosing cholangitis, porcelain gallbladder, gallbladder polyps or aflatoxin exposure, can raise the index of suspicion prior to surgery. However, these factors are often overlooked until after a pathologic diagnosis is made following a laparoscopic cholecystectomy for non-oncologic preoperative indications [10,11,12,13,14,15,16,17]. Even imaging obtained during the work-up of biliary disease provides few clues that distinguish between benign and malignant pathology. A single institution review of 5796 patients who had undergone cholecystectomy identified 26 patients (0.45%) with incidental GBC [18]. A blinded review of preoperative ultrasound images showed that gallbladder wall thickening without pericholecystic fluid was seen in 73.9% of GBC patients compared to 47.4% of non-GBC controls (*p* < 0.0001), and multivariate analysis revealed that patients with this finding were 3.5 times more likely to have incidental GBC. Nonetheless, these radiographic features, even when combined with other clinical features associated with higher risk of GBC, such as advanced age and elevated liver enzymes, are unlikely to alert general surgeons to a more insidious disease process and trigger a referral for oncologic evaluation for this rare diagnosis. Given the significant overlap in presentation with benign biliary disease, broad-sweeping but vague risk factors, and few reliable radiographic features, over half of all GBC is discovered incidentally during a non-oncologic index operation or on final pathology after surgical resection [19,20].

### 2.2. Surgical Challenges in Incidental Gallbladder Cancer

The incidental nature of GBC presents several obstacles for optimal treatment sequencing in the management of this devastating disease. This is made apparent by the fact that undergoing a non-oncologic index cholecystectomy for patients with incidental GBC is associated with worse long-term survival [21]. In a comparative review at two large hepatobiliary centers, patients with T2b GBC had a significantly worse 3-year survival when incidental GBC was diagnosed compared to when diagnosed preoperatively (31% vs. 85%; *p* = 0.019). Unsuspecting surgeons approach cholecystectomy for incidental GBC as if it were a difficult operation for cholecystitis, rather than an oncologic operation. This confounds surgical management by requiring a second operation for oncologic extended resection and pathologic staging. Moreover, surgery for benign biliary disease greatly diminishes the surgeon’s concern for gallbladder disruption at the index operation, a maneuver sometimes performed intentionally in cases of a difficult dissection or to define critical structures and aberrant anatomy, placing a patient at high risk for iatrogenic dissemination of malignancy [19,20]. Given the complexity of GBC, patients benefit from management at a specialized center with a multidisciplinary team including oncology, hepatobiliary surgery, gastroenterology, and interventional radiology. A national cancer database retrospective study investigating the difference in survival between academic and community treatment centers in the management of gallbladder cancer demonstrated a lower 30-day mortality (4.12% vs. 7.71%) and 90-day mortality (13.22% vs. 19.9%) and increased overall survival (13.6% vs. 11.0%) (*p* < 0.01) in a propensity score matched analysis [22]. However, in the event of required emergent cholecystectomy in the setting of concern for malignancy, histopathological examination of the specimen is essential and referral following resection would be appropriate. Following pathologic diagnosis, it is essential to obtain a complete staging workup prior to pursuing surgical or oncologic treatment. This should include, at minimum, contrasted cross-sectional imaging and diagnostic laparoscopy to rule out metastatic disease, according to an AHPBA expert consensus panel in 2015 [23]. The addition of positron emission tomography (PET) is also advocated by experts for completion imaging, given that metastatic disease would significantly change the approach to management. A 2019 meta-analysis found the sensitivity of PET scan in ruling out lymph node and distant metastases to be 88.4% (95% CI 82.6–92.8) and 85.4% (95% CI 79.5–90.2) [24]

Often, gallbladder cancer has an insidious onset with presentation of symptoms in late disease. Close monitoring of symptoms, such as pain and jaundice, may indicate more locally advanced disease involving the common bile duct. Further, development of these symptoms after oncologic resection may also suggest recurrence, triggering further investigation and the potential need for biliary decompression.

### 2.3. Impact of Bile Spillage on Recurrence Patterns

The 5-year survival for all patients with GBC is 18%, which decreases to 2% with metastatic disease [2]. A SEER-based analysis analyzing the prognostic value of site-specific metastases reported a median OS of 3 months with bone metastases, 4 months with liver metastases, 9 months with lung metastases, 6 months with distant lymph node metastases, and 4 months with multi-site metastases [25]. Multivariate analysis revealed that resection of the primary tumor in sub-populations with isolated liver and distant lymph node metastasis had better overall survival (OS) and cancer-specific survival (CSS). Most patients with distant metastases, other than isolated to the liver, have associated peritoneal disease linking peritoneal spread with poor outcomes in advanced disease [26]. Another review by Jarnigin et al. analyzing recurrence patterns following oncologic resection reported that of 55 patients with distant recurrence, peritoneal recurrence occurred in 25 patients (46%) [27]. Therefore, preventative strategies to limit peritoneal dissemination as part of standard oncologic management in non-metastatic GBC may lead to improved long-term outcomes.

Defining the features that place a patient with GBC at high risk for disease recurrence and peritoneal metastasis can help in determining who might benefit most from preventative treatment strategies such as prophylactic HIPEC. Two such factors supported by the literature are iatrogenic gallbladder perforation during surgery and the location of the primary tumor within the gallbladder. Spillage of gallstones and bile containing malignant cells can lead to seeding metastases onto the peritoneum [28]. Perforation rates during laparoscopic cholecystectomy for benign indications range from 10 to 30% and rates of stone spillage from 5.7 to 9% in several large institutional reviews [29,30,31,32]. Based on a nationwide survey organized by the Japanese Society of Biliary Surgery that included 253 hospitals, perforation during laparoscopic cholecystectomy for GBC occurred in 20% of 470 patients reviewed [33]. In a smaller series of 16 patients with T1 and T2 GBC, seven (44%) patients were found to have perforation with bile spillage [34]. Although the perforation rate reported is within the range or slightly higher than the rate seen with benign indications for cholecystectomy, the consequences of perforation and bile spillage can be far more consequential in GBC. In the Japanese survey, there was a significantly worse 5-year survival in patients with perforation compared to those without perforation (*p* < 0.01) and a trend toward increased metastatic recurrence in all sites reported, although not statistically significant [33]. One retrospective review by Goussous et al. including 17 cases of incidental GBC found that bile spillage led to a 2.6 times increased risk of peritoneal carcinomatosis (72.7% vs. 27.3%, *p* = 0.005) and those placed in this high-risk category strikingly had a median survival of 11 months compared to 45.5 months for patients without spillage (*p* = 0.005) [18]. Importantly, even patients with lower T stage seem to be at high risk when gallbladder disruption occurs, demonstrated by the fact that two patients with pT1 tumors in the nationwide Japanese survey died of peritoneal dissemination. Further, 50% of patients with bile spillage in the review by Goussous et al. had T2 tumors, likely contributing to the high rate of carcinomatosis and dismal survival outcomes reported [18,33].

The location of the primary tumor within the gallbladder is another factor influencing the risk of peritoneal spread. The unique anatomy of the gallbladder, with a portion juxtaposed to the liver and the remainder covered by peritoneum, influences the mode of metastasis and outcomes. Naturally, gallbladder tumors along the peritoneum surface have a higher risk of peritoneal dissemination compared to those along the hepatic wall. A cohort of 437 patients with GBC who underwent curative resection at four major hepatobiliary centers worldwide was reviewed, showing that the 5-year survival in patients with T2 GBC was 42.6% in hepatic-side tumors (*n* = 99) and 64.7% in peritoneal-side tumors (*n* = 153; *p* = 0.0006), with a similar, non-significant trend in survival in T1 tumors, 85% versus 90% (*p* = 0.61), respectively [35]. This insight led to an update to the 8th edition of AJCC TNM staging in which T2 was subdivided into T2a and T2b to reflect the prognosis of peritoneal-side versus hepatic-side tumors, respectively [36]. Once developed, peritoneal metastases are difficult to treat and result in significant morbidity and mortality. As a result, novel approaches that prevent or target peritoneal metastases are needed for this disease.

### 2.4. Cytoreductive Surgery and HIPEC for Peritoneal Metastases from Gallbladder Cancer

As with other biliary tract cancers, the current standard of care for patients presenting with metastatic GBC is palliative chemotherapy. Surgical indications in this setting are extremely limited and remain controversial; however, there is increasing evidence of a potential survival advantage when surgery is pursued. A population-based propensity-matched study comparing patients with distant metastatic GBC who underwent resection of the primary tumor (*n* = 496) versus no surgery (*n* = 841) revealed significantly higher OS (HR 0.62, 95% CI: 0.50–0.77, *p* < 0.001) and CSS (HR: 0.61, 95% CI: 0.50–0.76, *p* < 0.001) in the surgery group [37]. These results imply that decreasing the overall tumor burden by removing the primary tumor, even with unresected metastatic lesions, there is an associated survival benefit. Although this study did not report the proportion of patients who exhibited peritoneal dissemination, there are also several case reports showing CRS as part of a multimodal approach to patients with pathologically confirmed peritoneal metastasis who survived > 5 years [38,39]. In a multi-institutional investigation, completeness of cytoreduction scores of CC-0 or CC-1 (HR, 24.222; 95% CI, 2.008 to 5.5054; *p* < 0.001) was a prognostic factor for improved survival on multivariate analysis [40]. In a multi-institutional registry involving the Peritoneal Surface Oncology Group International, incomplete debulking surgery (completeness of cytoreduction [CCR], 2 or 3; *p* < 0.001) was an independent predictor of worse survival [41].

There are currently no consensus guidelines regarding the indications for HIPEC in the setting of gallbladder cancer peritoneal carcinomatosis prophylaxis. The use of HIPEC is considered experimental given the lack of sufficient research surrounding the benefits or lack thereof, but is reserved for peritoneal disease. Contraindications to HIPEC include allergy to cytotoxic chemicals, comorbid factors such as severe lung disease, cardiovascular disease, or hepatic and renal disease [42]. A Peritoneal Carcinomatosis Index (PCI) of 20 is the upper threshold of value, as the overall survival at 5 years for patients with PCI > 20 is approximately zero [43]. In a meta-analysis including 76 different studies, CRS and HIPEC in patients with colorectal peritoneal carcinomatosis had a mean mortality and morbidity of 2.8% (±2.9%) and 33.0 (±13.4%), respectively [44]. Complications of HIPEC include post-operative ileus, bone marrow suppression, infection, hepatic insufficiency, renal insufficiency (1.7%), and hematologic toxicity (5.6%) [45,46].

A retrospective case series reporting five patients who underwent CRS plus HIPEC for metastatic GBC with macroscopic peritoneal disease also showed compelling outcomes, reporting a median OS of 22.4 months and a 3-year survival of 30% [47]. One retrospective study published in 2018 reviewed the utility of CRS plus HIPEC in patients with biliary carcinomas and peritoneal metastases [9]. Using a collaborative international database, the authors compared CRS plus HIPEC (*n* = 34) to palliative gemcitabine and cisplatin therapy (*n* = 21). The authors found that both the median OS (21.4 vs. 9.3 months, *p* < 0.007) and the 3-year OS (30% vs. 10%) favored the CRS plus HIPEC group. Of note, this series, including only 55 patients over a period of 20 years, is the largest retrospective study published, demonstrating the paucity of data available and the difficulty of studying this patient population. Despite the lack of prospective data comparing CRS (with or without HIPEC), these studies suggest a survival advantage in patients with GBC with peritoneal metastasis, a patient population once thought not to benefit from surgery (CRS and primary tumor resection) or HIPEC.

### 2.5. Rationale for Prophylactic HIPEC

The studies above not only set a precedent for further investigation into the efficacy of CRS plus HIPEC for metastatic GBC, but also suggest an opportunity to study the application of these treatment strategies in patients without metastatic disease but at higher risk. The goal of HIPEC in the prophylactic setting is to treat microscopic peritoneal implants, thereby limiting the risk of peritoneal recurrence and improving patient outcomes after oncologic resection. The strategy of prophylactic HIPEC has been explored or is under active investigation for numerous gastrointestinal malignancies, including colon, gastric, and appendiceal cancers [3,4,5,6,7,8]. For example, building on the promising foundations set by cohort studies, many phase III RCTs studying prophylactic HIPEC in patients with colon cancer have recently been published, with several more currently being conducted (examples of active clinical trials: NCT02965248, NCT03914820, NCT04370925, NCT02974556) [5,7,8,48].

Data on the published RCTs are mixed, as two trials (PROPHYLOCHIP-PRODIGE 15 and COLOPEC) did not show a difference in their respective primary endpoints of 3-year disease-free survival and 18-month peritoneal metastasis-free survival, while a third trial (HIPECT4) did report a significant difference in its primary endpoint of 3-year locoregional control. Despite the negative results of PROPHYLOCHIP-PRODIGE 15 and COLOPEC, there are several important lessons that can be gleaned from these early trials in terms of future trial design. Both RCTs selected oxaliplatin-based regimens which may have diminished the efficacy of HIPEC because the standard-of-care systemic therapy containing oxaliplatin (given to 89% of patient in PROPHYLOCHIP-PRODIGE 15) causes chemoresistance to intraperitoneal oxaliplatin. A higher concentration of oxaliplatin was required to kill tumor cells when a patient received systemic treatment within 2 months in an ex vivo study [49]. Furthermore, the duration of perfusion was only 30 min in both RCTs rather than the typical 60–90 min treatment time, which limits the penetration of cancer with chemotherapy. Another important pattern to note when comparing these trials is the trend in the proportion of patients who have macroscopic peritoneal disease during a second-look operation when HIPEC administration is intended as a prophylactic rather than therapeutic treatment. The PROPHYLOCHIP-PRODIGE 15 trial performed a second-look operation after 6 months of adjuvant treatment and found that 52% of patients had macroscopic peritoneal disease that was radiographically occult. The COLOPEC trial had a cohort that received “adjuvant” HIPEC 6–8 weeks after upfront surgery in order to include patients requiring emergent surgery for obstruction or perforation, scenarios in which HIPEC is contra-indicated at the time of surgery. Only 10% of these patients in the COLOPEC trial had macroscopic peritoneal disease on second look. These findings suggest that the optimal timing for administration of HIPEC prophylactically is during simultaneous oncologic resection, if possible. This approach might benefit those who would go on to develop peritoneal disease even within a short interval time. Further, earlier administration of HIPEC may also maximize the penetration of tumor deposits as adhesive disease from a previous operation may exclude certain areas of the peritoneum from chemotherapy. Caution should be taken when interpreting these data, as the treatment populations, regimens, and surgery performed were different between trials, making comparison difficult. In contrast to these important RCTs, the HIPECT4 trial is a phase III multi-center RCT that found a significant difference in the primary outcome measure of 3-year locoregional control [8]. Briefly, patients with locally advanced colon cancer (cT4N0-2M0) were randomized to CRS (including tumor resection, lymphadenectomy, omentectomy, appendectomy, ± oophorectomy) with or without heated MMC for 60 min. The 3-year locoregional control in the HIPEC group was 97.6%, versus 87.6% in the surgery-alone arm (log-rank *p =* 0.03; HR 0.21; 95% CI, 0.05–0.95). No differences were seen in the secondary outcomes of 3-year disease-free survival or 3-year OS. Important differences in this study compared to the PROPHYLOCHIP-PRODIGE 15 and COLOPEC trials include a more homogenous treatment population, and the use of MMC with a longer perfusion time of 60 min. Additionally, all patients in the HIPEC treatment group received the treatment at the time of the index oncologic resection, which eliminates the morbidity of a second operation and the potential to miss the treatment window before peritoneal dissemination occurs. There was no difference between the groups in the post-operative complications seen when HIPEC was combined with CRS, supporting the safety and feasibility of this approach. As critical prospective data continue to accumulate in colon cancer populations, appropriate indications and optimal regimens for prophylactic HIPEC will become progressively clearer.

As noted above, the low prevalence of GBC relative to other cancers, such as colon and gastric, makes robust prospective randomized trials nearly impossible to conduct. Unfortunately, this makes the existing prospective data extrapolated from other cancers and the, albeit scarce, retrospective data in GBC the highest level of evidence available to support the use of prophylactic HIPEC in GBC. The early success of prophylactic HIPEC in other gastrointestinal malignancies offers hope that a similar strategy may be efficacious in non-metastatic GBC. Indeed, a retrospective review in patients with stage III GBC published in 2021 shows an associated benefit with the addition of HIPEC as a multimodal treatment strategy [50]. This study compared the control group (radical surgery and capecitabine; *n* = 43) to the HIPEC group (radical surgery plus HIPEC and capecitabine; *n* = 35). The authors report a median survival of 19.2 months versus 15.3 months favoring the HIPEC group and a significant difference in 1-year and 2-year survival rates between the two groups (91.4% vs. 76.7% and 26.2% vs. 17.5%, respectively, *p* < 0.05).

Assessment of adverse effects and perioperative outcomes after implementing an experimental intraoperative treatment is critical. In the above study, the days of hospitalization were significantly different in favor of the control group (20.0 ± 5.8 vs. 23.0 ± 6.9 days, *p* = 0.029) [50]. There was no significant difference in post-operative complications, including when stratified based on Clavien-Dindo classifications (I/II and III/IV). Post-operative biochemical data were not different except for a decreased platelet count in the HIPEC group, which did not reach the level of thrombocytopenia (233.1 ± 79.0 vs. 296.7 ± 75.8 × 10^9^/L, *p* = 0.001). While there was also no difference in the rate of infection between treatment groups, the rate of infection is still notably high (34%) in the HIPEC group. The increased risk of infection following CRS plus HIPEC is a known phenomenon in other cancers. In a retrospective review of CRS plus HIPEC for colorectal cancer and pseudomyxoma peritonei, infectious complications were reported to occur in 43% of patients with surgical site infections occurring in 27% of all patients [51]. The use of immunosuppressive medications combined with complex surgical intervention makes an increased risk of infection unsurprising. The promise of achieving a significant survival advantage with the addition of prophylactic HIPEC outweighs the differences seen in the perioperative outcomes published in this retrospective study.

### 2.6. Prospective Clinical Trial of Prophylactic HIPEC in Gallbladder Cancer

Incidentally discovered GBC is treated with hepatic IVb and V segmentectomy and regional lymphadenectomy; therefore, a prophylactic HIPEC can be easily incorporated into the second operation performed as part of the standard of care. We have initiated a single institution, prospective clinical trial of prophylactic HIPEC for cases in which there is a pathologically proven GBC by prior pathology or intra-operative frozen section and inadvertent spillage of bile or intentional decompression during cholecystectomy or tumors extending through the serosa of the gallbladder (T3/T4) (Clinicaltrials.gov registration #NCT05430035). HIPEC is performed at the time of the index operation if a cancer is known pre-operatively, or at the time of re-operation for incidentally discovered cancers already treated with cholecystectomy that are undergoing hepatic resection and regional lymphadenectomy as part of the standard of care. Minimally invasive and open approaches for HIPEC, as indicated by the liver resection/lymphadenectomy, are both being studied. We have planned to utilize a closed technique with 30 mg of MMC over the first 60 min followed by 10 mg over an additional 30 min. The perfusate is heated to obtain a tissue temperature of 42 °C. In this pilot trial, we will evaluate the safety and feasibility of prophylactic HIPEC for GBC, with the goal of obtaining data in support of a larger, multi-institutional trial powered to reduce the risk of peritoneal recurrence and improve overall survival.

### 2.7. Future Directions

Surveillance following oncologic resection of GBC consists of cross-sectional imaging every 3–6 months with or without obtaining tumor markers such as CEA and CA 19-9 [52]. Despite this, the current cross-sectional imaging used to identify peritoneal metastases in multiple cancers remains inadequate. This is well demonstrated in the PROPHYLOCHIP-PRODIGE 15 trial described above, by the fact that 52% of patients deemed to have an absence of peritoneal disease on CT scan were found to have macroscopic disease at the time of surgery [7]. One strategy that may supplement imaging, and other clinical indicators of prognosis, is the application of artificial intelligence (AI) models that increase the sensitivity and specificity of GBC prognosis and recurrence prediction, thereby directing appropriate treatment strategies. One study applied an AI model “avNNet” to two series of multi-clinical indicators made up of independently validated prognostic indicators in GBC [53]. By applying this model to clinical data from 122 patients, the authors were able to show an area under the curve (AUC) of 0.944, sensitivity of 86%, and specificity of 100% for predicting prognosis in one series and an AUC of 0.882, sensitivity of 77%, and specificity of 100% in the other. These multi-clinical indicators were applied to Kaplan–Meier analysis, showing excellent prediction of disease-free survival and overall survival. Future application of such AI models may have a role in prognostication in GBC.

Another frontier in oncology that may help to distinguish patient subgroups at the highest risk of metastasis and peritoneal spread as well as GBCs sensitive to biologic and/or immunotherapies is the application of genetic sequencing data. Exciting and practice-changing advances are rapidly occurring across the field that take a personalized approach to treating advanced and aggressive cancers [54]. Briefly, examples include the use of biologics to target CLDN18.2 expression in gastric cancer to stimulate antibody dependent cytotoxicity, applying novel switch-control tyrosine kinase inhibitors in patients with GISTs with specific *KIT* exon mutations, the combination of pembrolizumab plus trastuzumab in HER2 overexpressed advanced gastric cancer, and the use of PARP inhibitors in pancreatic cancer patients with pathogenic variants *BRCA* and *PALB2* [55,56,57,58,59,60]. One study analyzed the mutational signatures associated with histopathology and prognostic biomarkers of biliary tract cancers and found 19 genes proposed as potentially actionable targets (PATs) [61]. In 46 patients with treatment refractory cancers who received PAT-matched therapies, an objective response rate was seen in 26.1% and the progression-free survival was 5 months. While current strategies rely on clinical and pathologic factors, a deeper biologic understanding with next-generation sequencing data may unveil distinct tumor subtypes associated with specific patterns of dissemination.

## 3. Conclusions

GBC may develop as an occult neoplasm, and subsequently, the malignancy may be discovered incidentally or in advanced progression. The late discovery, unexpected presentation, and potential for complications during resection contribute to the dismal prognosis. Here, we have outlined the epidemiological magnitude and risk factors for peritoneal dissemination of GBC. We report a case of successful administration of HIPEC administered with prophylactic intent, and the initiation of a prospective trial utilizing HIPEC with prophylactic intent against GBC-associated peritoneal disease. The relatively infrequent nature of gallbladder cancer impedes successful clinical trials on this disease. While we anticipate accruing to our prospective feasibility trial, a randomized trial demonstrating the efficacy of prophylactic HIPEC in preventing peritoneal dissemination will require a multi-institutional effort.
